# A Case of Maturity-Onset Diabetes of the Young With Complex Mutations

**DOI:** 10.1210/jcemcr/luae031

**Published:** 2024-03-16

**Authors:** Sheetal Bulchandani, Priya Kundra

**Affiliations:** Bon Secours Southside Medical Center, Petersburg, VA 23805, USA; Department of Endocrinology, Medstar Washington Hospital Center, Washington, DC 20010, USA

**Keywords:** MODY, *HNF4A*, *PAX4*

## Abstract

Maturity-onset diabetes of the young (MODY) encompasses a group of rare monogenic forms of diabetes, with 14 subtypes described in the literature, each with a distinct underlying genetic mutation. We present a case with mutations in 2 different genes that are known to be responsible for MODY. A 33-year-old male individual presented to the endocrinology clinic for evaluation. He was diagnosed with type 2 diabetes mellitus at 13 years of age and was initially treated with insulin, which was subsequently switched to repaglinide and metformin. The patient reported a history of hypoglycemia at birth and in his daughter. His biological father was diagnosed with diabetes mellitus at 16 years of age. Genetic testing for monogenic diabetes revealed a pathogenic variant in hepatocyte nuclear factor 4 alpha and a variant of unknown significance in Paired Box Gene 4. The treatment was switched to glipizide 2.5 mg orally, which resulted in adequate glycemic control. Genetic testing was recommended for his daughter. MODY can be missed because of its broad clinical presentation. Heightened vigilance and a low threshold for genetic testing for MODY are required in patients with a high likelihood of having MODY, as the treatment can be tailored to individual patient needs.

## Introduction

Maturity-onset diabetes of the young (MODY) comprises several monogenic forms of diabetes that are distinct in etiology, pathophysiology, and clinical presentation from the more common forms of type 1 (autoimmune) and type 2 diabetes. First described in 1975, the estimated prevalence of MODY ranges from 1% to 5% among all forms of diabetes. There are at least 14 subtypes of MODY with different genetic mutations, each affecting the beta cell function in a different way [1]. Here, we present a unique case with mutations in 2 different genes that are known to be responsible for 2 different forms of MODY, namely, hepatocyte nuclear factor 4 alpha (*HNF4A*) and Paired Box 4 (*PAX4*).

## Case Presentation

A 33-year-old male individual with a past medical history of type 2 diabetes mellitus presented to the endocrinology clinic for evaluation. He was diagnosed with type 2 diabetes mellitus at 13 years of age and was initially treated with insulin, which was subsequently switched to repaglinide and metformin. At the time of presentation, he was taking metformin 1000 mg orally twice a day and repaglinide 0.25 mg orally with each meal 2 to 3 times per day. His family history was significant for a diagnosis of type 2 diabetes mellitus in his father at 16 years of age. He reported a history of hypoglycemia at birth and reported that his recently born daughter was hypoglycemic at birth. Physical examination revealed a body mass index of 22 kg/m^2^. Other examination findings were unremarkable.

## Diagnostic Assessment

The laboratory parameters on presentation are presented in Table 1. Islet cell cytoplasmic antibody testing, and glutamic acid decarboxylase (GAD) 65 antibody testing results were negative. Genetic testing was performed by Medical Neurogenetics LLC (MNG)/LabCorp. Genes assessed by this test included *ABCC8*, *APPL1*, *BLK*, *GCK*, *HNF1A*, *HNF1B*, *HNF4A*, *INS*, *KCNJ11*, *KLF11*, *NEUROD1*, *PAX4*, and *PDX1*. Genetic testing revealed a c.893-2A > G heterozygous mutation in the eighth exon region of the *HNF4A* gene, resulting in an adenine to guanine substitution within the acceptor splice site for exon 8, and an intronic variant c.563-5C > T in the fifth exon region of the *PAX4* gene, leading to a cytosine to thymine substitution.

**Table 1.** Laboratory evaluation on presentation

Parameter	Result	Reference range
HbA1c	5.9%	4.8%–5.6%
LDL	95 mg/dL (2.46 mmol/L)	< 99 mg/dL (< 2.56 mmol/L)
HDL	51 mg/dL (1.32 mmol/L)	40–59 mg/dL (1.03–1.53 mmol/L)
Triglycerides	193 mg/dL (2.18 mmol/L)	0–149 mg/dL (0–1.68 mmol/L)
Urine microalbumin/creatinine ratio	6 mg/dL (0.53 mmol/L)	0–30 mg/dL (0–3.39 mmol/L)
Serum creatinine	0.75 mg/dL (66.32 mmol/L)	0.56–1.00 mg/dL (65.4–119.3 mmol/L)

Abbreviations: HbA1c, hemoglobin A1C; HDL, high-density lipoprotein; LDL, low-density lipoprotein.

Genomic regions of interest were selected using a custom capture reagent for target enrichment (Twist Bioscience) and sequenced using the Illumina Nova6000 next-generation sequencing platform. Sequencing reads were aligned with the human genome reference GRCh37/hg19 build. The regions of interest included all exons and intron/exon junctions (+/− 20 nucleotides) for each gene analyzed.

Next-generation sequencing data were used to call single nucleotide polymorphisms (SNPs), and small indels were assessed using the Illumina's Dynamic Read Analysis for GENomics (DRAGEN) Bio-IT platform. Genes listed in ClinVar with intragenic pathogenic deletions were padded with additional intronic probes to allow single exon resolution copy number variation (CNV) detection.

The patient was heterozygous for both mutations. The mutation in *HNF4A* gene was labeled as a pathogenic variant, and the mutation in *PAX4* was reported as a variant of unknown significance as per the American College of Medical Genetics and Genomics (ACMG) guidelines.

## Treatment

Results of the genetic testing were discussed with the patient, and his treatment regimen was switched to glipizide 2.5 mg orally daily.

## Outcome and Follow-Up

A follow-up hemoglobin A1C (HbA1c) test 3 months later revealed an HbA1c of 5.6%. *HNF4A* mutation testing was performed for the patient's daughter and son, with positive results for both.

## Discussion

MODY is a rare monogenic form of diabetes, differing from type 1 and type 2 diabetes in etiology, pathophysiology, and clinical presentation. Various subtypes of MODY have been described in several publications by both their underlying genetic mutations as well as numerical designation (ie, MODY 1 to MODY 14) [1].

HNF4A MODY, also known as MODY 1, is the third most common form of MODY overall, with a reported prevalence ranging from 5% to 10% among all forms of MODY.

The *HNF4A* gene is located on chromosome 20, and its transcription is activated by the *HNF1A* gene. In turn, the *HNF4A* protein regulates the expression of the *HNF1A* gene.

Patients with an *HNF4A* mutation may have decreased high-density lipoprotein cholesterol (HDL-C) levels and are therefore like patients with type 2 diabetes mellitus in that regard. In addition, these patients have a higher birth weight and a higher level of macrosomia. They may display transient neonatal hypoglycemia [2]. However, our patient had neonatal hypoglycemia and a normal birth weight and no macrosomia.

PAX transcription factors, defined by the conserved bipartite paired box DNA-binding domain (PAIRED), are classified into 4 groups according to the presence/absence of an octapeptide region and presence/absence/truncation of a second DNA-binding homeodomain. *PAX4* is predominantly expressed in pancreatic β- and δ-cells and helps in their differentiation and in maintaining beta cell function [3]. A mutation in *PAX4* can disrupt the PAX4 protein targeting ability, increase the number of alpha cells, and increase glucagon secretion, resulting in elevated blood glucose [4]. A mutation in the *PAX* gene causes MODY 9. MODY 9 was first described in 2 patients of Thai origin who did not present with mutations in the other known MODY genes, and since then, several case reports have been described [4-6]. In MODY 9, missense mutations or frameshift deletions have been reported, and patients can be homozygous or heterozygous for the mutation [5]. The estimated frequency of the *PAX4* gene mutation in the general population is as low as 0.0001, which indicates that it is a low-frequency mutation [6].

The molecular diagnosis of MODY may dictate the choice of the most appropriate treatment, with the aim to optimize blood glucose control, reduce the risk of hypoglycemic events and long-term complications, and enable proper genetic counseling. Sulfonylureas are typically recommended for patients with MODY 1 and often for patients with MODY 9. Some patients may require insulin [7].

Given its varied clinical presentation, patients with MODY may be misdiagnosed as possessing another form of diabetes, resulting in potentially inappropriate treatment and delays in the screening of affected family members and associated comorbidities. Our case highlights the importance of maintaining high vigilance and a low threshold for genetic testing for MODY in patients who have a high likelihood of having MODY, as the treatment can be changed as per the presentation of each patient.

## Learning Points

- MODY encompasses monogenic forms of diabetes that are distinct from type 1 and type 2 diabetes, with varying prevalence rates (1%–5%).
- Patients with MODY may present diverse clinical profiles due to mutations in different genes. Consequently, it is crucial to be aware and have a proactive approach, with a low threshold for genetic testing.
- Identifying the specific genetic mutations can aid in tailoring treatment.

## Contributors

All authors made individual contributions to authorship. S.B. was involved in the writing of the manuscript and submission. P.K. was involved in the diagnosis and treatment of the patient, case selection, and obtaining consent. All authors reviewed and approved the final draft.

## Data Availability

Data sharing is not applicable to this article as no datasets were generated or analyzed during the current study.

## Abbreviations

HbA1c: glycated hemoglobin
MODY: maturity-onset diabetes of the young
